# Outcomes of Cytoreduction and Oxaliplatin-Based Hyperthermic Intraperitoneal Chemotherapy in Patients With Peritoneal Carcinomatosis From Colorectal Cancer

**DOI:** 10.7759/cureus.18670

**Published:** 2021-10-11

**Authors:** Moayad Alhumaid, Salma Sait, Emad Fallatah, Nasser AlSayegh, Ali Farsi, Mohammed Nassif, Nada J Farsi, Nouf Akeel, Ali Samkari, Alaa A Shabkah, Nora Trabulsi

**Affiliations:** 1 Department of Surgery, King Abdulaziz University Faculty of Medicine, Jeddah, SAU; 2 Department of Dental Public Health, King Abdulaziz University Faculty of Dentistry, Jeddah, SAU; 3 Department of Surgery, International Medical Center, Jeddah, SAU

**Keywords:** oxaliplatin, cytoreductive surgery and hipec, cytoreductive surgery (crs), peritoneal carcinoma, colorectal neoplasm, systematic review and meta-analysis

## Abstract

Among patients with metastatic colorectal cancer, 25% have isolated peritoneal carcinomatosis. We performed a systematic review and meta-analysis to assess the disease-free survival (DFS) and overall survival (OS) of patients undergoing hyperthermic intraperitoneal chemotherapy with oxaliplatin. Eleven studies were included in the final assessment. Pooled three- and five-year OS rates were 58.60% and 42.19%, respectively. The estimated pooled three- and five-year DFS rates were 23.47% and 14.26%, respectively.

## Introduction and background

Worldwide, colorectal cancer (CRC) is the second most common cancer in women and the third most common in men. The global incidence of CRC in 2018 accounted for 10.2% of all cancers [1]. Peritoneal carcinomatosis (PC) affects around 30% to 40% of patients with CRC, 5% to 10% of whom will have the synchronous disease [2,3]. It is the sole metastasis site in approximately 25% of patients [4]. Although patients presenting with PC arising from CRC previously received palliative treatment, in carefully selected patients, it is no longer considered a terminal condition [5]. In the past few decades, such patients have instead been managed with cytoreductive surgery (CRS) and hyperthermic intraperitoneal chemotherapy (HIPEC) [6-13].

CRS plus HIPEC has become a promising management modality that improves disease-free survival (DFS) and overall survival (OS) rates in PC secondary to CRC. This modality can yield five-year OS rates of 19% to 51%, which in the case of palliative systemic treatment is only 13% [5,7]. Approaching a locoregional disease is best achieved by locoregional modalities [14]. The rationale behind this treatment modality is to remove all macroscopic diseases with CRS and microscopic cells by HIPEC [15]. A major advantage of HIPEC is the exposure of the malignant cells to a hyperthermic chemotherapeutic agent, thus enhancing its activity and increasing the toxicity to neoplastic cells [16-18].

Multiple cytotoxic agents can be used for HIPEC as intraperitoneal (IP) chemotherapy. As there is no solid evidence for the superiority of one chemotherapeutic agent over another in PC arising from CRC, the decision about which chemotherapeutic agent to use is based on multiple variables, including, but not limited to, the tumor histology and individual experience at the PC treatment center [5]. Mitomycin C (MMC) and oxaliplatin are currently the most commonly used IP chemotherapeutic agents for HIPEC. Compared with systemic infusion, in HIPEC, the high molecular weights of oxaliplatin and MMC permit their exposure to malignant cells in high concentrations and to prompt their absorption [19,20]. Studies that compared these two agents reported varying results regarding survival benefits [5,21-24]. More recently, surgeons have used IP irinotecan in combination with oxaliplatin, but with no improvement in OS or DFS [8].

Despite oxaliplatin being the drug of choice in systemic adjuvant and palliative settings [25], the survival benefit in the setting of HIPEC has not been compared with other treatment modalities in randomized settings. Whether the use of oxaliplatin in HIPEC provides additional survival benefit to patients is controversial, given the preliminary results of the PRODIGE 7 and the PROPHYLOCHIP trials [26,27]. Non-randomized data suggest that oxaliplatin may be superior to other IP agents.

In this systematic review and meta-analysis, we assessed studies for the survival benefit of patients with CRC and PC who underwent treatment with CRS plus HIPEC in which oxaliplatin was used.

## Review

Protocol registration

The protocol for this systematic review is available at http://www.crd.york.ac.uk/PROSPERO/. The registration code is CRD42017069766.

Search strategy

A comprehensive literature search was performed from March to July of 2018 in PubMed, Web of Science, and Google Scholar electronic databases. We used the following Medical Subject Headings (MeSH) terms when searching PubMed: (“Oxaliplatin” [Supplementary Concept] OR “Antineoplastic Agents” [Mesh]) AND “Colorectal Neoplasms” [Mesh] AND “Peritoneal Neoplasms” [Mesh]. Animal studies were excluded.

Study selection

Studies were screened by title and/or abstract. Reviewers assessed the retrieved articles for inclusion and exclusion criteria, and independently assessed all of the articles. The senior author resolved any disagreements between reviewers.

Inclusion and exclusion criteria

For a study to be considered, it had to include primary CRC patients with PC who underwent CRS plus HIPEC, with oxaliplatin being used in at least one of the HIPEC groups. It also had to be published in English and to report OS and/or DFS for the oxaliplatin group. We excluded case reports, review studies, phase I clinical trials, non-human studies, studies that included pediatric patients (<18 years of age), pharmacokinetics and dynamics studies, studies in which the primary tumor origin was other than colorectal, studies of pressurized IP aerosol chemotherapy, studies of non-oxaliplatin-based HIPEC, and overlap in patients/data between studies. If the same trial was reported several times, we used the most recent publication to avoid replication of study participants.

Quality assessment

The methodological index for non-randomized studies (MINORS) criteria were used to assess the quality of the studies in this systematic review and meta-analysis [28]. Three authors (MA, SS, and EF) assessed the quality individually, which was then reviewed by NT for any disagreement. The cutoff for an acceptable quality assessment score for non-comparative studies was set at 75% (equivalent to 12 points) and for comparative studies at 75% (18 points).

Data extraction

Three authors (MA, SS, and EF) independently extracted data from the included studies by using a predesigned extraction form created by the most senior author, containing the following: author, publication year, setting, the total number of patients in the study, number of patients receiving oxaliplatin, the overall male proportion of patients and male proportion of patients receiving oxaliplatin, median and mean age of patients overall and those receiving oxaliplatin chemotherapeutic agents used for HIPEC, comparison chemotherapeutic agent (for either HIPEC or systemic use), median DFS and OS for the oxaliplatin group, DFS and OS overtime for the oxaliplatin group, and median follow-up period. Three authors (MA, SS, and EF) reviewed the extracted data for further validation of values and minimization of error during the extraction process. After data extraction, the authors of the included papers were contacted to provide additional data that might help to expand the systemic review. However, most of the authors did not respond.

Statistical analyses

The summary estimates of the one-, two-, three-, and five-year DFS and OS rates were calculated after applying weights that were based on the sample size of the individual studies. Random-effects models were used in the presence of heterogeneity, while fixed-effects models were used in their absence [29]. Forest plots were created to graphically represent the summary estimates and the contributions of the individual studies.

Heterogeneity among the studies was assessed by the index of heterogeneity (I^2^) [30,31]. This test statistic is presented in percentage form, expressing the proportion of variation across the different publications attributed to heterogeneity [30]. The lower the number, the less the heterogeneity present. Publication bias was assessed by visually inspecting Begg’s funnel plots for five-year DFS and five-year OS rates and by Egger’s regression asymmetry tests [29].

P-values of ≤ 0.05 indicated statistically significant results. All statistical significance tests were two sided, and the analyses were performed by using Stata 12.1 (StataCorp LP, College Station, TX, USA).

Results

Study Selection and Literature Search

**Figure 1:**
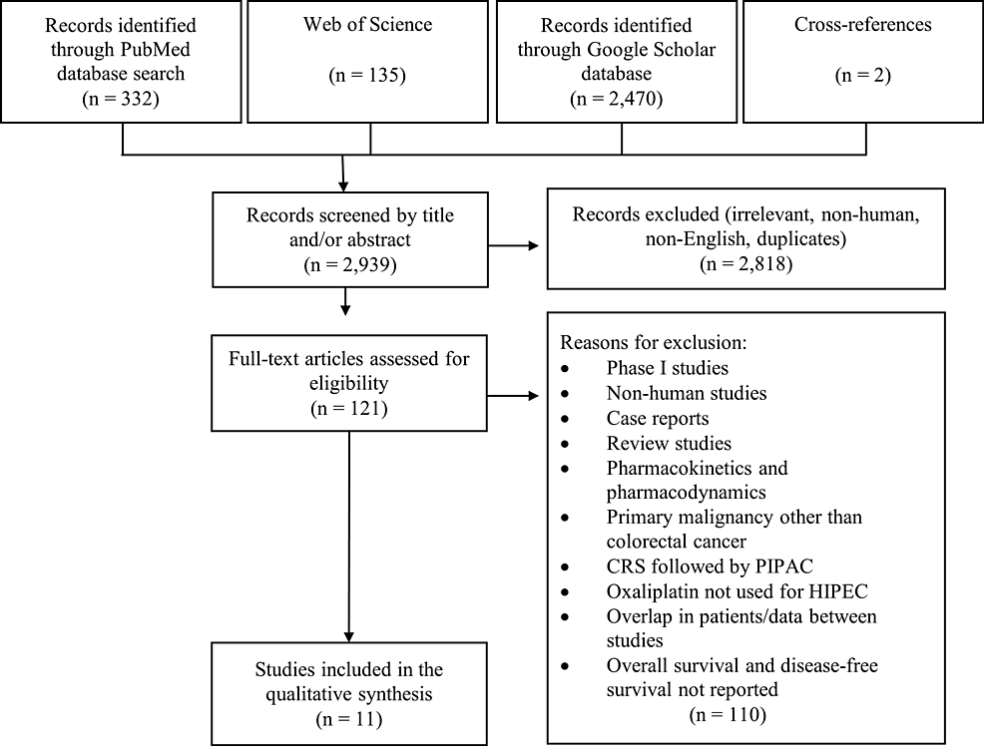
PRISMA flow diagram of study identification, screening, eligibility, and inclusion. HIPEC: hyperthermic intraperitoneal chemotherapy; PIPAC: pressurized intraperitoneal aerosol chemotherapy; PRISMA: Preferred reporting items for systematic reviews and meta-analyses.

In total, 2,939 articles were identified through the databases (Figure 1). First these studies were assessed by title and/or abstract. Of these studies, 2,818 were excluded because they were irrelevant, not English, non-human, or duplicates. The initially included studies narrowed down to 121. After a full-text review of these studies, 11 were included in the final quantitative synthesis (Table 1).

**Table 1:** Summary of the trials included in the meta-analysis Ø = Not mentioned in the study. § = The rest of the cohort had primaries other than colorectal cancer. Abbreviations: CRC = colorectal cancer; MMC = mitomycin

Author/ Publication year	Setting	Study population size	Number of patients administered oxaliplatin	Male proportion (oxaliplatin group), %	Median or (mean) age, years (oxaliplatin group)	Comparison group	Disease-free survival (oxaliplatin group)	Overall survival (oxaliplatin group)	Median follow-up, months	Quality score	PCI Mean / Median
Cavaliere et al., 2011 [22]	Multicenter, Italy	146	11	51.4 (overall)	56 (overall)	Cisplatinum or MMC	Ø	Ø	19 (overall)	12/16	Ø
Elias et al., 2009 [7]	France	96	48	64	(46)	Systemic chemotherapy	Ø	2 years = 81% 5 years = 51%	63	20/24	Ø
Faron et al., 2016 [32]	France	173	173	41	48.9	None	5 years = 14%	5 years = 42%	48.5	12/16	Mean = 10.2 (6.8) Median = 11
Gervais et al., 2013 [33]	Canada	40	25	57.5 (overall)	56.5 (overall)	None	3 years = 22%	3 years = 61% 5 years = 36%	22.8	12/16	Median = 10
Glockzin et al., 2014 [34]	Germany	32	20	45	(53)	Irinotecan	Ø	2 years = 70% 3 years = 65%	39.4 (overall)	13/24	Mean overall = 13 Mean oxaliplatin group = 13 Mean Irinotecan group = 12
Hompes et al., 2012 [35]	Multicenter, Belgium	48	48	35.4	60	None	1 year = 65.8 2 years = 45.5	1 year = 97.9% 2 years = 88.7%	22.7	10/16	Median = 11
Leung et al., 2017 [36]	Australia	202	96	54.2	(55.5)	MMC	Ø	Ø	Ø	15/24	Mean overall = 9.4 (6.3) Mean oxaliplatin group = 8.8 (5.3) Mean MMC group = 10 (7.1)
Prada-Villaverde et al., 2014 [5]	Multicenter, North America and Europe	584	166	55.4	(56.9)	MMC	Ø	Ø	Ø	13/24	Ø
Quenet et al., 2011 [8]	Multicenter, France	146	43	Oxaliplatin 27.9, oxaliplatin + irinotecan, 46.6	51.5 (overall)	Oxaliplatin + irinotecan	5 years oxaliplatin group = 13.8% 5 years oxaliplatin + irinotecan group = 14.2%	5 years oxaliplatin group = 41.8% 5 years oxaliplatin + irinotecan group = 42.4%	48.5 (overall)	20/24	Median = 11
Turrini et al., 2012 [37]	France	60	26^§^	33.3 (overall)	(52.1) (overall)	None	(Primary CRC) 1 year = 42% 3 years = 25% 5 years = 20%	1 year = 100% 3 years = 51% 5 years = 37%	41	12/16	Mean = 9.6 (4.2)
Van Leeuwen et al., 2008 [38]	Sweden	103	38	46.6 (overall)	55 (overall)	None	Ø	2 years = 63.7	13	11/16	Median = 22

Study Characteristics

An outline of the studies included in this analysis, with their references, is displayed in Table 1. A total of 11 studies were included in the meta-analyses. The publication years ranged between 2007 and 2017. Of the 11 studies, seven were prospective [7,8,22,32,35,37,38] and four were retrospective [5,33,34,36]. None of the included studies were randomized controlled trials (RCTs). Across all studies, 797 of 1,630 patients underwent HIPEC with oxaliplatin. Among these studies, three included the addition of irinotecan to oxaliplatin in HIPEC (294 patients) [7,8,32]. The remaining 833 patients were excluded because they received HIPEC chemotherapeutic agents other than oxaliplatin (MMC, cisplatinum, or irinotecan).

The mean or median age of the patients is presented in Table 1. The proportions of male patients ranged between 33.3% and 65.6%. The median follow-up time ranged from 13 to 63 months. Patients in seven of the included studies received neoadjuvant treatment [7,8,32-34,37,38]. All included studies used a regimen based on oxaliplatin only, except for the study by Faron et al, which used oxaliplatin plus irinotecan [32]. When present, for comparison, the second arm of the studies varied between using HIPEC based on non-oxaliplatin only [5,8,22,34,36] or systemic chemotherapy [7]. Other HIPEC agents based on non-oxaliplatin only included MMC [5,22,36], cisplatinum [22], and irinotecan alone [34] or combined with oxaliplatin [8]. Seven studies were performed in a single center [7,32-34,36-38] and four were multicentric [5,8,22,35]. Four studies took place in France [7,8,32,37] and the rest were distributed among Italy [22], Belgium [35], Sweden [38], Australia [36], Canada [33], and Germany [34]; one took place in North America and Europe [5].

Primary Outcomes

Five studies reported the DFS over time [8,32,33,35,37], only one of which reported the 95% confidence interval (CI) of the DFS over time [35]. Eight studies reported the OS over time [7,8,32-35,37,38], only two of which reported the 95% CI of the OS over time [7,35].

**Figure 2:**
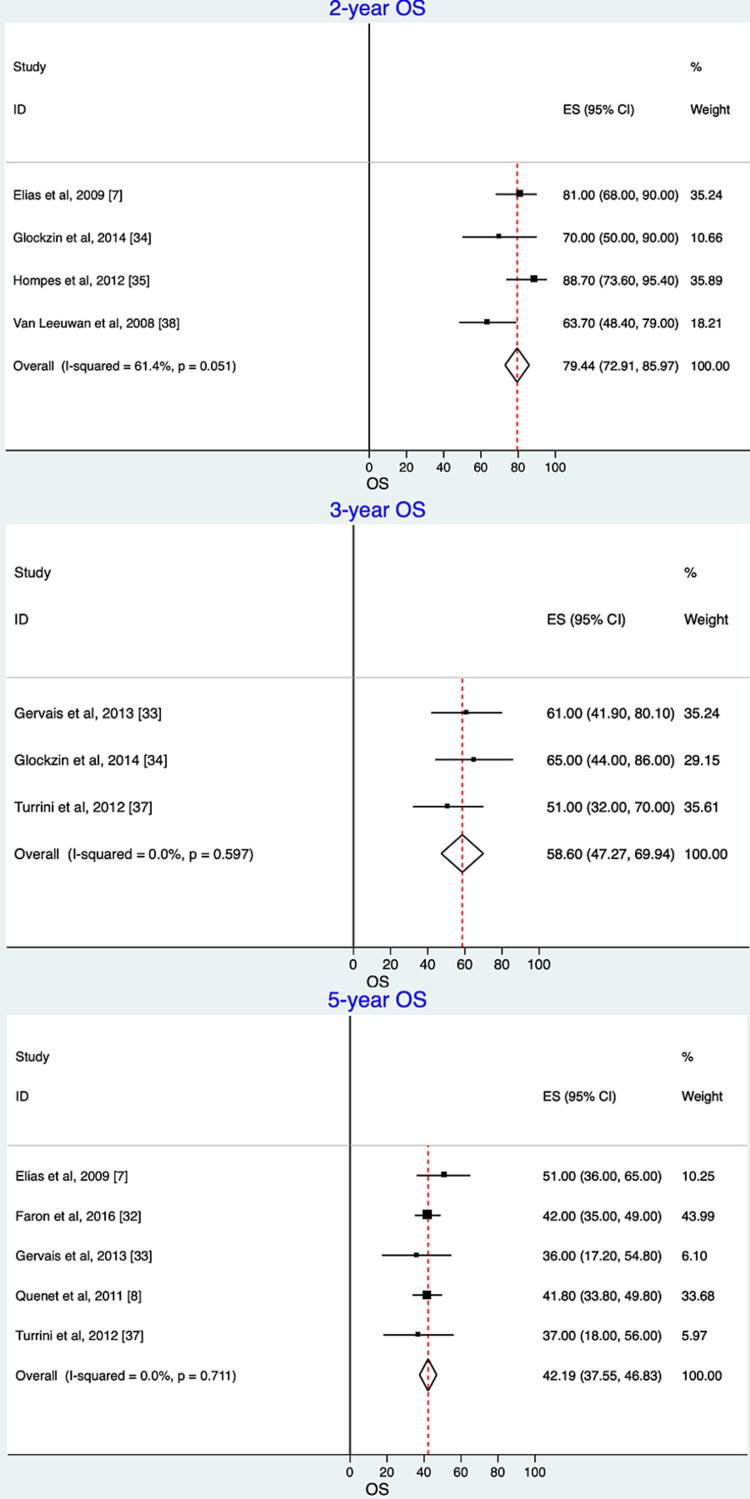
Overall survival (OS) over time for oxaliplatin-based hyperthermic intraperitoneal chemotherapy. Meta-analysis and forest plots of OS along with summary estimates and 95% confidence intervals (CIs). The studies are listed in alphabetical order. The horizontal lines represent 95% CI for each study. The squares represent the estimates, with their sizes proportional to the statistical weight of each. The vertical vertex of the diamond illustrates the summary overall survival estimate. The sides of the diamond illustrate 95% CI. ES: effect size Forest plot of two-year OS estimates: Elias et al., 2009 [7], Glockzin et al., 2014 [34], Hompes et al., 2012 [35], Van Leeuwen et al., 2008 [38]. Forest plot of three-year OS estimates: Gervais et al., 2013 [33],  Glockzin et al., 2014 [34], Turrini et al., 2012 [37]. Forest plot of five-year OS estimates: Elias et al., 2009 [7], Faron et al., 2016 [32], Gervais et al., 2013 [33], Quenet et al., 2011 [8], Turrini et al., 2012 [37].

Not all studies reported one-, two-, three-, and five-year survival estimates. We present here the summary estimate of the OS and DFS for the studies that reported them. The pooled estimates of two-, three-, and five-year OS are presented in Figure 2. The two-year OS of patients receiving oxaliplatin for HIPEC plus CRS was 79.44% (95% CI: 72.91-85.97; I^2^ = 61.4%; P = 0.05) for those studies that reported the two-year OS. The three-year pooled estimate for OS was 58.6% (95% CI: 47.27-69.94; I^2^ = 0.0%; P = 0.59), while the five-year OS was 42.19% (95% CI: 37.55- 46.83; I^2^ = 0.0%; P = 0.71) for those studies that reported the two-, three-, and five-year OS rates.

**Figure 3:**
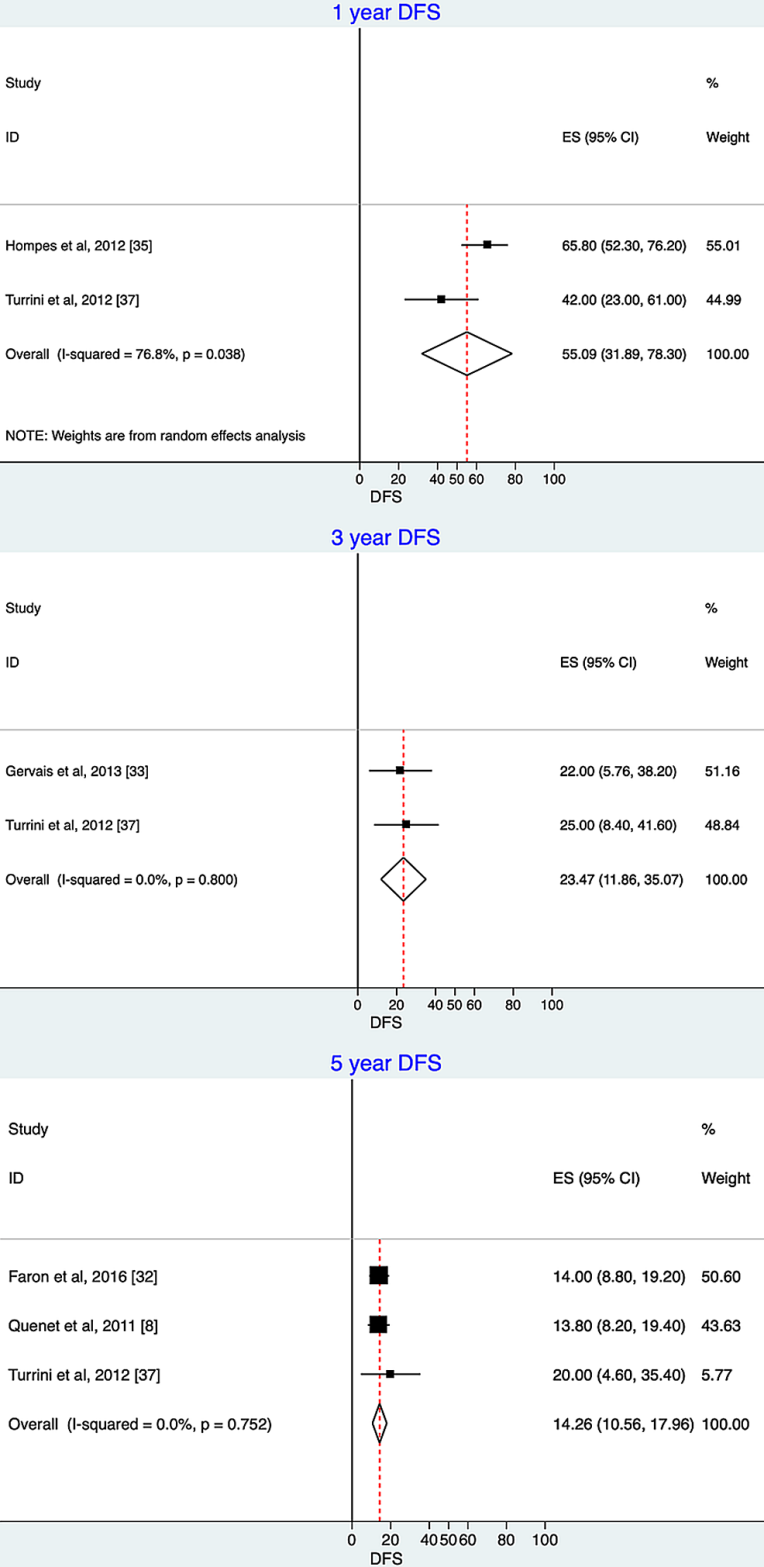
Disease-free survival (DFS) over time for oxaliplatin-based hyperthermic intraperitoneal chemotherapy. Meta-analysis and forest plots of DFS along with summary estimates and 95% confidence intervals (CIs). The studies are listed in alphabetical order. The horizontal lines represent 95% CI for each study. The squares represent the estimates, with their sizes proportional to the statistical weight of each. The vertical vertexes of the diamonds illustrate the summary disease-free survival estimates. The sides of the diamonds illustrate 95% CI. ES: effect size Forest plot of one-year DFS estimates: Hompes et al., 2012 [35], Turrini et al., 2012 [37]. Forest plot of three-year DFS estimates: Gervais et al., 2013 [33], Turrini et al., 2012 [37]. Forest plot of five-year DFS estimates: Faron et al., 2016 [32], Quenet et al., 2011 [8], Turrini et al., 2012 [37].

The one-year DFS was 55.09% (95% CI: 31.89-78.3; I^2^ = 76.8%; P = 0.03), the three-year DFS was 23.47% (95% CI: 11.86-35.07; I^2^ = 0.0%; P = 0.80), and the five-year DFS was 14.46% (95% CI: 10.56-17.96; I^2^ = 0.0%; P = 0.75) for those studies that reported one-, three-, and five-year DFS rates (Figure 3).

Three studies included in our systematic review assessed the addition of irinotecan to oxaliplatin [7,8,32]. The total number of patients across these studies was 294. The five-year OS rates reported by Elias et al. [7], Faron et al. [32], and Quenet et al. [8] were 51%, 42%, and 42.4%, respectively. The five-year DFS rates reported by Faron et al. [32] and Quenet et al. [8] were 14% and 14.2%, respectively, whereas this rate was not reported by Elias et al. [7].

Study Quality

Using MINORS criteria for the quality assessment of the included studies, we found that the median for the comparative studies [5,8,34,36,38] was 15/24 with a range of 13-20, with only 40% achieving a score of 75% or more. On the other hand, the median score in the non-comparative studies [7,22,32,33,35,37] was 12/16 with a score ranging from 10 to 12, with 66.7% achieving a score of 75% or higher.

Publication Bias

**Figure 4:**
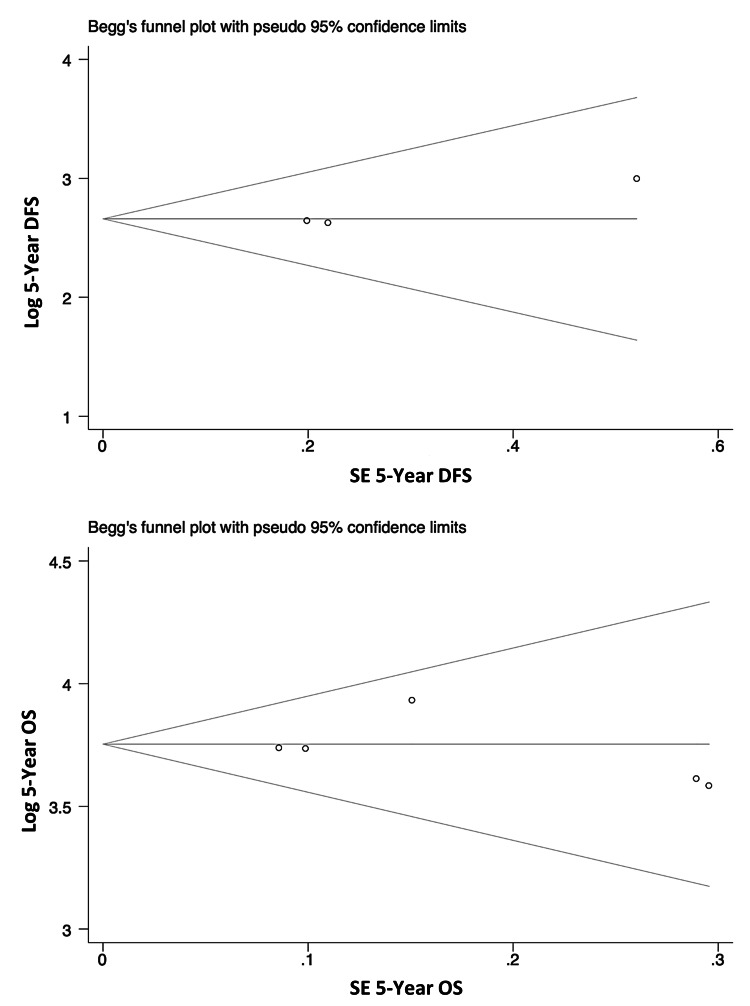
Begg’s funnel plot for the assessment of publication bias in 5-year disease-free survival (DFS) and 5-year overall survival (OS) rates. The horizontal line illustrates the fixed-effects summary estimate, while the sloping lines indicate the expected 95% confidence intervals for a given standard error (SE).

Begg’s funnel plots for the five-year DFS and five-year OS rates are shown in Figure 4. No publication bias was observed.

Discussion

With medical and surgical advancements, PC in selected patients with primary CRC is no longer incurable, and surgical treatment is gaining acceptability. Before the HIPEC era, survival rates with traditional systemic chemotherapy for patients with PC were between 5.2 and 12.6 months [16].

During our literature search, no randomized clinical trials matched our inclusion criteria. This probably relates to the fact that PC from CRC is a complex disease that is just beginning to be understood. Our systematic review calculated the pooled estimate of survival rates for patients who underwent HIPEC with oxaliplatin. In our study, the two-, three-, and five-year OS rates were 79.44% (95% CI 72.91-85.97), 58.6% (95% CI 47.27-69.94), and 42.19% (95% CI 37.55- 46.83), respectively. The one-, three-, and five-year DFS rates were 55.09% (95% CI 31.89-78.3), 23.47% (95% CI 11.86-35.07), and 14.26% (95% CI 10.56-17.96), respectively.

No standardized HIPEC regimen has been proven to have a superior survival outcome compared with other regimens. Some centers use MMC as an agent of choice and others prefer oxaliplatin. As oxaliplatin is one of the agents of choice for systemic treatment [39], logic suggests that it is superior to MMC in HIPEC for CRC. No previous meta-analysis has been conducted, however, that compares oxaliplatin head to head with MMC. We aimed in this systematic review to contribute to this debate by assessing oxaliplatin survival outcomes.

Oxaliplatin is suggested as initial therapy for metastatic CRC in patients who are not candidates for CRS plus HIPEC [39]. In patients who are found to be sensitive to platinum-based chemotherapy, it has been reported that repeated administration of oxaliplatin continued to show a systemic response [40,41]. In animal models, local infusion of oxaliplatin in the peritoneal cavity results in a high local concentration with minimal systemic absorption and toxicity [42].

PRODIGE 7 is a phase III RCT that compares CRS plus HIPEC with oxaliplatin against seldom CRS. The preliminary results showed the benefit of oxaliplatin-based HIPEC in patients with a mid-range (11-15) PC index (PCI), as opposed to no benefit for patients with a low-range PCI (<11) [26]. Our meta-analysis did not assess the effect of PCI on the DFS and OS of patients receiving HIPEC with oxaliplatin. Another RCT with recently released preliminary results is the PROPHYLOCHIP trial. It evaluated the benefit of prophylactic oxaliplatin-based HIPEC in patients with CRC at high risk of PC. Early results showed no difference in outcome for patients at high risk of developing PC undergoing prophylactic HIPEC compared with that of controls [27]. Our opinion is that select patients with a peritoneal recurrence may still benefit from oxaliplatin-based HIPEC.

Huang et al. [43] conducted a systematic review of PC secondary to CRC. Their primary endpoint was an evaluation of the efficacy and effectiveness of HIPEC in CRC. In seven studies, 614 patients received MMC-based HIPEC. Four studies with a total of 283 patients received oxaliplatin-based HIPEC. For MMC-based HIPEC, the one-, three-, and five-year OS was 79.5%, 38.8%, and 34%, respectively. For oxaliplatin-based HIPEC, the one-, three-, and five-year OS was 93%, 59%, and 43%, respectively. These findings are consistent with the OS of our systemic review and meta-analysis of our 11 included studies and 797 patients.

Postoperative complications in HIPEC include hematological complications related to the therapeutic agents, which have been reported to occur in 13.6% of patients [44]. One of the arguments against the use of oxaliplatin-based HIPEC is that, compared with MMC, it is associated with more such complications [45]. The literature on this is inconclusive. In a retrospective study, Votanopoulos et al. [46] compared toxicities between oxaliplatin and MMC in the setting of HIPEC. Oxaliplatin had statistically significant grade 3 or 4 platelet toxicity. Their study also demonstrated a trend toward a significantly higher grade 2-4 neutrophil toxicity [46]. Other studies demonstrated that MMC is associated with a higher number of neutropenic toxicities [21]. Another aspect to consider when using oxaliplatin-based HIPEC is that it has been shown to be an independent risk factor for hemorrhagic complications in the postoperative period [45,46].

Our systematic review has several limitations. None of the included studies were RCTs. Regarding the quality of the included studies, five studies scored less than 75% based on the MINORS criteria. Five studies did not mention whether there was any loss of follow-up, which could contribute to attrition bias. We believe that the other items in the MINORS score do not directly influence the validity of our results. We did not assess the role of PCI on survival in this systematic review, as it was not available in all included studies, which is increasingly thought to be a major factor in this population. Another limitation is that the included studies did not report the same survival outcomes. Results of publication bias should be interpreted with caution, as there were a few studies assessed in the funnel plot. There was a low response rate from the study authors who we contacted in order to obtain survival data. Our study was limited to the English language, which may contribute to language bias.

Our study’s strengths are that, to our knowledge, it is the largest systematic review and meta-analysis to assess survival for oxaliplatin-based HIPEC in PC secondary to CRC. Our pooled estimates also had low heterogeneity among the included studies, making the summary estimates more reliable. Moreover, our results are consistent with the previously published literature on this topic.

## Conclusions

This study supplies healthcare providers with a summary of survival results associated with the use of oxaliplatin-based HIPEC in the literature. Having these estimates aids clinicians and their patients in deciding the appropriateness and benefit of oxaliplatin-based HIPEC. The limited number of good-quality prospective RCTs may affect the results. Moreover, assessment of the effect of PCI on DFS and OS of patients receiving oxaliplatin-based HIPEC is likely to help determine the population that would benefit from this regimen. Finally, definitive conclusions await an RCT that compares oxaliplatin-based HIPEC versus MMC-based HIPC versus CRS alone.
